# Supplementary material for: Data-driven head motion correction for PET using time-of-flight and positron emission particle tracking techniques
Source: PLoS One. 2022 Aug 31;17(8):e0272768. doi: 10.1371/journal.pone.0272768 (PMC9432725; doi:10.1371/journal.pone.0272768)
Supplement: Attachment — Submitted filename: ReviewerResponse.docx [file pone.0272768.s001.docx]

Point by Point Response to Reviewers

1. Point source dosimetry not included in manuscript

Point source dosimetry information was added to the discussion section

*The proximity of the point sources warranted some concern for possible exposure to the sensitive lens of the eye even though the point sources used in this work were very weak. As part of our initial IRB review and approval, we estimated lens doses to the eye under worst-case conditions using a 5 microCurie source positioned 1 cm directly in front of the eye without accounting for beta shielding from the plastic goggles. Our total dose rate estimated under these worst case conditions was 14.3 mGy/hr which even for 30 minutes of total exposure would be 70x less than even the updated 0.5 Gy deterministic limit set by the International Commission on Radiation Protection in 2018. Our more realistic, yet still conservative, estimates assuming a 5 microCurie source, goggle beta shielding, 1 cm distance, and 15 minutes of exposure resulted in a total estimated dose of 0.07 mGy to the lens of the eye.*

2. Independent Goggle Movement from Patient head

Additional information on the goggles and our testing was added to our goggles/glasses discussion in the discussion section.

*We tested different surfaces on which the point sources can be placed including a head band and directly on the skin but found lab goggles to be more comfortable for the patient while also providing a secure fit. Using a structure to separate the source from the patient skin is desirable as it enables an air gap between the activity naturally being taken up by the patient tissue and the sources being tracked. For this study, the patients were told when to move so we knew when to visually observe for any possible glasses movement. Additionally, the ranges of motion assessed also did not result in head positioning that caused contact with the goggles that would have caused unwanted movement and would have resulted in an additional PET acquisition. Only visual assessment of motion was performed but we did not observe movement of the glasses relative to the head for any of the patients, however, one limitation of using external sources that are not attached to the skin would be the potential risk for the glasses to shift during imaging in such way as to no longer be aligned with the patient head movement.*

3. Consent information

Additional information was added to the methods section related to patient consent location and to highlight the approved IRB protocol related to this study.
